# Special Tributes

**DOI:** 10.1007/s11538-021-00956-7

**Published:** 2021-12-03

**Authors:** James D. Murray

**Affiliations:** 1 Wolfson Centre of Mathematical Biology, University of Oxford, Oxford, UK; 2 Department of Applied Mathematics, University of Washington, Seattle, USA

It was with great sadness that I learned of the death of Nick Britton during the preparation of this Special Collection. Nick was one of my first PhD students in mathematical biology at Oxford, graduating five years before I established the Centre for Mathematical Biology. Nick went on to a long and distinguished academic career, spent mostly at the University of Bath, which founded its own Centre for Mathematical Biology in 2004, with Nick as Co-Director. His research work was wide ranging, and he made important contributions to mathematics applied in many areas of the life sciences, including ecology, epidemiology, evolution, cancer, artificial kidneys and pain—the last of these in collaboration with his wife Suzanne Skevington. In addition to his many research papers, Nick wrote two highly influential books—“Reaction-diffusion Equations and their Applications to Biology” and “Essential Mathematical Biology”—which are key reference texts and teaching tools in our community.

Nick was a longtime member of the Society for Mathematical Biology and served on its Board of Directors from July 2006 until July 2010, and was Chair of the Future Meetings Committee from July 2008—July 2010. But Nick’s contribution to mathematical biology was much greater than this. He was an inspirational figure, a dedicated teacher and a great listener, who quietly but effectively influenced the academic trajectory of many students and colleagues. We will miss him.

James D. Murray, January 2021

Very sadly, Masayasu Mimura, known to his friends as Mayan, who had accepted the invitation to contribute to this Special Collection, passed away on April 8th, 2021. He came to Oxford in 1976 and was my first postdoc and, while working with me, his interests extended from understanding the qualitative aspects of reaction-diffusion systems to exploring their behaviour in the context of mathematical biology, and we published a number of papers together. He then moved back to Japan and started a research group in mathematical biology at Hiroshima University. Mayan went on to become a very influential figure in Japan. He held many leadership positions, including Director of the Institute for Nonlinear Sciences and Applied Mathematics (INSAM),

Faculty of Science, Hiroshima University (1998–2004), overlapping with his time as Chairman of the Department of Mathematical and Life Sciences, Graduate School of Science, Hiroshima University (2000–2001). He then went on to Meiji University, where he was Director of the Meiji Institute of Advanced Study of Mathematical Sciences (2007–2015). He was President of the Japanese Society of Industrial and Applied Mathematics (JSIAM), President of the Japanese Society of Mathematical Biology, and he held many visiting positions.

Mayan had a truly original mind and he advanced the field of reaction-diffusion theory in many different directions. For example, he developed a global bifurcation theory for reaction-diffusion systems in the context of biological and ecological applications, proposed the singular limit procedure in the context of species competition models, worked on free boundary problems in ecology and developed novel models for patterning in bacterial colonies. He had a warm personality, an infectious enthusiasm for his science and was very supportive and encouraging to young researchers. He will be sorely missed.

James D. Murray, May 2021

